# Integrating transcriptomics, eQTL, and Mendelian randomization to dissect monocyte roles in severe COVID-19 and gout flare

**DOI:** 10.3389/fgene.2024.1385316

**Published:** 2024-09-25

**Authors:** Jiajia Li, Guixian Yang, Junnan Liu, Guofeng Li, Huiling Zhou, Yuan He, Xinru Fei, Dongkai Zhao

**Affiliations:** ^1^ Changchun University of Chinese Medicine, Changchun, Jilin, China; ^2^ Third Affiliated Clinical Hospital to Changchun University of Chinese Medicine, Changchun, Jilin, China

**Keywords:** COVID-19, gout, mendelian randomization, monocyte, single-cell RNA sequencing

## Abstract

**Introduction:**

There are considerable similarities between the pathophysiology of gout flare and the dysregulated inflammatory response in severe COVID-19 infection. Monocytes are the key immune cells involved in the pathogenesis of both diseases. Therefore, it is critical to elucidate the molecular basis of the function of monocytes in gout and COVID-19 in order to develop more effective therapeutic approaches.

**Methods:**

The single-cell RNA sequencing (scRNA-seq), large-scale genome-wide association studies (GWAS), and expression quantitative trait loci (eQTL) data of gout and severe COVID-19 were comprehensively analyzed. Cellular heterogeneity and intercellular communication were identified using the scRNA-seq datasets, and the monocyte-specific differentially expressed genes (DEGs) between COVID-19, gout and normal subjects were screened. In addition, the correlation of the DEGs with severe COVID-19 and gout flare was analyzed through GWAS statistics and eQTL data.

**Results:**

The scRNA-seq analysis exhibited that the proportion of classical monocytes was increased in both severe COVID-19 and gout patient groups compared to healthy controls. Differential expression analysis and MR analysis showed that *NLRP3* was positively associated with the risk of severe COVID-19 and involved 11 SNPs, of which rs4925547 was not significantly co-localized. In contrast, *IER3* was positively associated with the risk of gout and involved 9 SNPs, of which rs1264372 was significantly co-localized.

**Discussion:**

Monocytes have a complex role in gout flare and severe COVID-19, which underscores the potential mechanisms and clinical significance of the interaction between the two diseases.

## 1 Background

The 2019 coronavirus (COVID-19) pandemic was an unprecedented global health crisis, and is expected to remain the leading cause of infection-related deaths in the coming years (Nasrullah et al., 2023). Most patients infected with the novel coronavirus present with mild or moderate symptoms, and only a small proportion progress to severe disease. The heterogeneity of outcomes highlights the necessity for a deeper understanding of COVID-19 pathogenesis. Gout is a crystal-associated arthropathy caused by monosodium urate (MSU) deposition in the joints, which manifests as recurrent pain, limited joint movement or leads to deformities and gouty nephropathy in severe cases. During the COVID-19 pandemic, gout patients experienced an increased frequency of acute attacks and elevated urate levels (Garcia-Maturano et al., 2022). Furthermore, gout increased the likelihood of positive COVID-19 diagnosis by 1.5 times (Topless et al., 2021).

The severe acute respiratory syndrome coronavirus 2 (SARS-CoV-2) can trigger a pro-inflammatory state by activating inflammasomes in infected monocytes and macrophages (Junqueira et al., 2021). Hyperuricemia, the key pathological factor of gout, can induce monocyte-associated inflammation and induce the production of chemokine ligand 2 and other inflammatory factors, leading to an increase in the immune response to secondary stimuli (Kluck et al., 2023; Li et al., 2023; Tanaka et al., 2017). COVID-19 and gout can occur simultaneously, which could exacerbate the symptoms by inducing systemic inflammation, immune cell overactivation, and inflammatory cytokine storm.

COVID-19 and gout share several risk factors, such as age, metabolic syndrome, and underlying disease. In addition, the severity of COVID-19 is highly dependent on host factors, and gout is caused by combination of genetic and environmental factors (Tin et al., 2019; Zhang et al., 2020). Studies show that innate immune cells, particularly monocytes, play a key role in driving the progression of gout and severe COVID-19. However, the exact role of monocytes in the interaction between the two diseases has not been fully elucidated. Due to the recent advances in genomics, transcriptomics, proteomics, and metabolomics, it is now possible to explore the pathogenesis of various diseases at the molecular level, and identify functional changes associated with disease progression. Furthermore, Mendelian randomization (MR) can overcome the limitations of observational studies and provide new insights into disease etiology and treatment by using genetic variants as instrumental variables. In this study, we integrated RNA sequencing (RNA-seq) data and genome-wide association studies (GWAS) data to explore the molecular basis of monocyte function in severe COVID-19 and gout flare.

## 2 Methods

### 2.1 Bioinformatics data sources

RNA-seq datasets were retrieved from the Gene Expression Omnibus (GEO) database. GSE192391 included the scRNA-seq data for severe COVID-19; GSE211783 included the scRNA-seq data of six samples from three gout patients. GSE157103 consisted of the bulk RNA-seq data of 50 severe COVID-19 patients and 26 healthy individuals.

### 2.2 GWAS and eQTL data sources for MR and colocalization analysis

**FIGURE 1:**
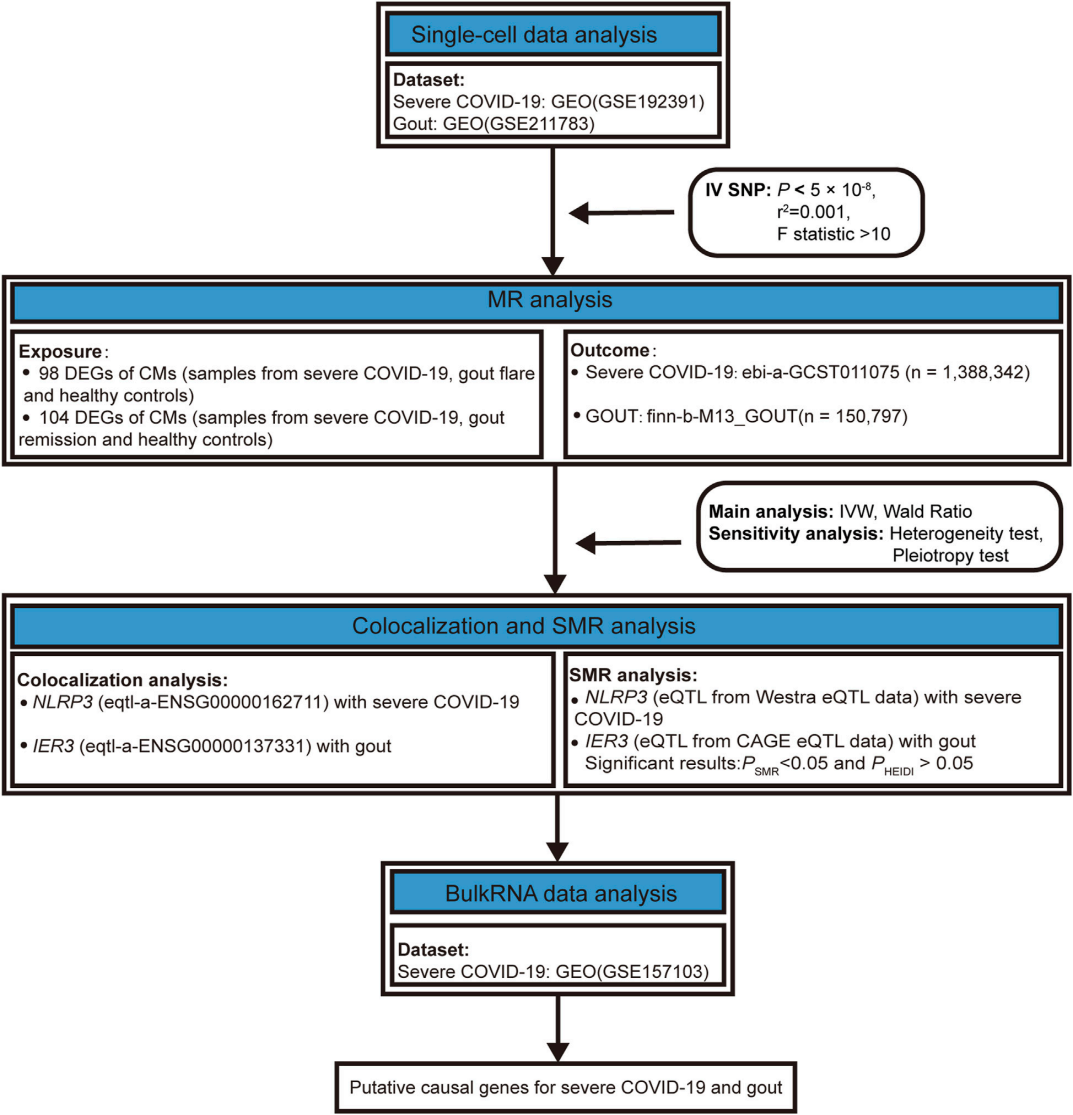
The summary of the study design.

In this study, we performed MR Analyses by using the R software package TwoSampleMR to identify single nucleotide polymorphisms (SNPs) associated with severe COVID-19 and gout, which were defined as instrumental variables. The GWAS data were obtained from the website (http://gwas.mrcieu.ac.uk/datasets), including the severe COVID-19 dataset (ebi-a-GCST011075, n = 1,388,342) and the gout dataset (finn-b-M13_GOUT, n = 150,797). The expression quantitative trait loci (eQTL) data were also publicly available from the IEU OpenGWAS project accession code eQTL-a-ENSG00000162711 and eQTL-a-ENSG00000137331. Our study design was summarized in Figure 1.

### 2.3 Single-cell data analysis

Quality control of datasets was performed using the Seurat package (version 4.4.0) in R software (version 4.3.1). Samples were first converted to Seurat objects using the “CreateSeuratObject”. Cells with mitochondrial gene percentages < 25% and unique gene counts between 200 and 6,000 were used. The data were normalized using the “NormalizeData” function, scaled for all genes using the ScaleData function, and subjected to principal component analysis (PCA). Hypervariable genes were identified by the FindVariableFeatures function and used for downstream analysis. Since the data were obtained from different samples, batch correction was performed using the R package “Harmony” (1.0.3) to avoid any batch effects interfering with downstream analysis. Cell clustering and classification were performed by using the FindClusters function. The SingleR package (2.2.0) was then used to match the single-cell RNA-seq data to a known reference dataset and manually calibrated to improve the accuracy and reliability of cell type annotation. Marker genes are shown in Supplementary Figures S1A, S1B. Monocyte subpopulations were categorized as classical monocytes (*CCR2*, *SELL*, *S100A8*, *S100A9*, *LYZ*, *SERPINB2*, *CD14*), non-classical monocytes (*NAP1L1*, *FCGR3A*, *FCGR3B*, *CSTA*, *CX3CR1*, *ITGAL*), and intermediate monocytes (*HLA-DRA*, *HLA-DPB1*, *EVL*) based on markers genes listed in literature. Dot plots show the proportion and average expression of cell clusters expressing the marker gene in monocytes from COVID-19, gout flare and healthy samples (Supplementary Figure S1C), and the marker gene in monocytes from COVID-19, gout remission and healthy samples (Supplementary Figure S1D). The correspondence between cell clusters, cell marker and cell types from COVID-19, gout flare and healthy samples are shown in Supplementary Figure S2A, and cell types from COVID-19, gout remission and healthy samples are shown in Supplementary Figure S2B. The FindAllMarkers function was used to find differentially expressed genes between monocyte and other clusters. The communication between monocytes and other cells was determined by analyzing the ligand-receptor pairs using CellChat (version 1.6.1) R package, with CellChatDB.human as the reference database. The R package scMetabolism was used to quantify the metabolic activities of the different types of cells at the single-cell level.

### 2.4 eQTL MR analysis

To ensure the robustness and reliability of the findings, we followed the STROBE-MR guidelines and used a two-sample MR approach (Skrivankova et al., 2021). Two sample MR analysis was performed by using the TwoSampleMR package (0.5.7), with genes as the exposure and the disease as the outcome. Variants in multiple regions of the genome were selected as instrumental variables in this study. The significantly associated SNPs (*P* < 5 × 10^−8^) were screened, and the threshold for removing linkage disequilibrium (LD) was set to r^2^ < 0.001. The instrumental strength for each SNP was assessed using the F statistic, with an F statistic >10 indicating a strong tool. The F statistic was calculated using the formula: $F=\frac{R^{2}\times \left(N-2\right)}{1-R^{2}}$ , where $R^{2}=\frac{2\times \beta^{2}\times \text{EAF}\times \left(1-EAF\right)}{2\times \beta^{2}\times \text{EAF}\times \left(1-\text{EAF}\right)+2\times {\text{SE}}^{2}\times N\times \text{EAF}\times \left(1-\text{EAF}\right)}$ (Papadimitriou et al., 2020; Levin et al., 2020; Gill et al., 2019; He et al., 2024). For genes with only one instrumental variable (IV), the Wald ratio method was used; otherwise, the inverse variance weighting method (MR-IVW) was used. In this study, *P* = 0.00052 (0.05/96) after correction of the Bonferroni method. Therefore, *P* < 0.00052 was considered significant.

### 2.5 Co-localization analysis

We performed a Coloc test to examine the probability that SNPs associated with disease and gene expression (eQTL) are shared genetic causal variants. Colocalization analyses were performed respectively by using eqtl-a-ENSG00000162711 as exposure data, ebi-a-GCST011075 as outcome data, and eqtl-a-ENSG00000137331 as exposure data and finn-b-M13_GOUT as outcome data. The instrumental variables were determined based on 1 MB upstream and downstream of the SNP with the lowest *p*-value associated with gene. Coloc package is used in R to evaluate Bayesian factors under various colocalization assumptions. Based on Bayesian statistical modeling, five posterior probabilities were generated, corresponding to the five hypotheses described by Wallace et al. Two hypotheses were the focus of this study: 1) PPH3, association with the severe COVID-19 or gout risk and expression of the gene, with distinct causal variants; 2) PPH4, association with the severe COVID-19 or gout risk and expression of the gene, with a shared causal variant. Posterior probabilities (PP) were used to quantify support for all hypotheses, and co-localization analyses were restricted to genes that achieved PPH3+PPH4 ≥ 0.8 (Giambartolomei et al., 2014). Finally, we obtained the genotype data of the target genes including eqtl-a-ENSG00000162711 and eqtl-a-ENSG00000137331, which were used to drawing the regional associations plots.

### 2.6 SMR analysis

Summary-data-based Mendelian randomization (SMR), based on Mendelian randomization, was proposed in 2016 by Zhu et al. (2016). The SMR analysis used single nucleotide variants of the top cis-eQTL as instrumental variables and combined GWAS and eQTL data to detect associations between gene expression and traits. In this study, we used the default settings in the SMR software. The *P*-value threshold for selecting the relevant eQTL for the SMR test is 5.0 × 10^−8^, and the window around the probe center for selecting cis-eQTL is 1 Mb. The eQTL data were obtained from two sources. Respectively, we used a study conducted by Westra et al. (Westra et al., 2013), which was an eQTL meta-analysis of peripheral blood samples from 5,311 healthy European individuals, and CAGE eQTL whole blood expression data, including 2,765 subjects of European ancestry (Lloyd-Jones et al., 2017). The criteria of significant results were as follows: *P*_SMR_<0.05 and *P*_HEIDI_ > 0.05.

## 3 Results

### 3.1 Cell clustering and annotation

**FIGURE 2:**
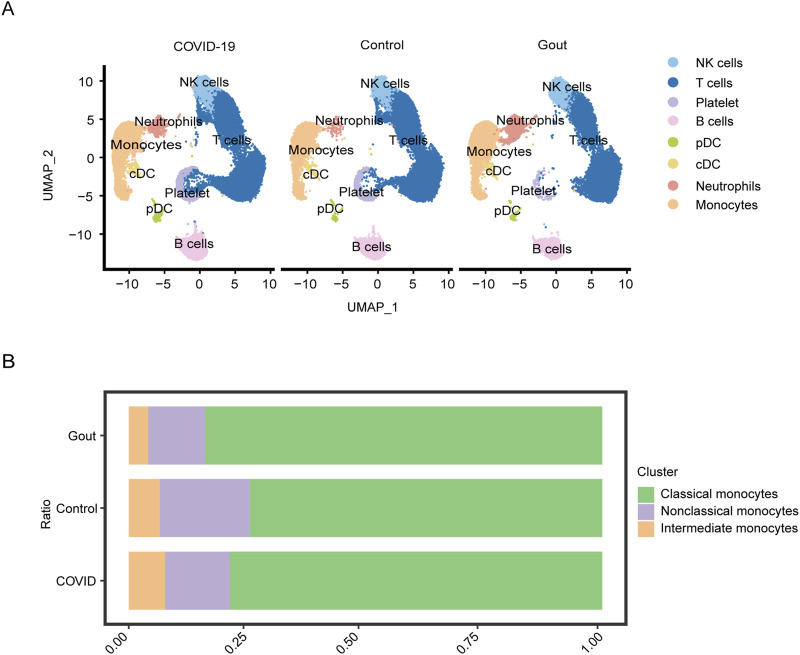
Single-cell analysis of severe COVID-19, gout flares and healthy samples. **(A)** UMAP plot displays the results after clustering. NK cells, T cells, B cells, pDCs, cDCs, monocytes, neutrophils, and platelets were identified. **(B)** Depicts the proportion of each monocyte subgroup in different samples.

In this study, we focused on the relationship between severe COVID-19 and gout flare. The scRNA-seq data of PBMCs obtained from patients with severe COVID-19, gout flare, and healthy samples revealed significant differences in the cellular composition of different groups. Based on specific gene markers, PBMCs were clustered into 20 distinct subclusters. Six clusters (clusters 13, 14, 15, 17, 18, 19) were considered “mixed” as the marker genes were not associated with any common or specific cell type, and were subsequently excluded due to the small number of cells. The final cell clusters including natural killer (NK) cells, T cells, B cells, plasmacytoid dendritic cells (pDCs), classical DCs (cDCs), monocytes, neutrophils, and platelets, and the UMAP plot is shown in Figure 2A. The monocytes were analyzed further given their critical role in gout and severe COVID-19 progression. Based on the expression of marker genes, we identified seven distinct monocyte clusters, including classical monocytes (CMs; clusters 0, 1, 4, and 6), non-classical monocytes (NCMs; clusters 2 and 5), and intermediate monocytes (IMs; cluster 3). As shown in Figure 2B, the proportion of classical monocytes was higher in gout flare and severe COVID-19 samples compared to the healthy controls. The differentially expressed genes (Supplementary Table S1) in the monocytes included those related to cytokines (*IL1B*, *NFKBIA*, *NLRP3*), inflammation (*CCR1*), cell adhesion (*JAML*), and apoptosis (*IER3*, *MCC1*). Similar to the above results, scRNA-seq dataset from patients with severe COVID-19 and during gout remission were analyzed, and the distribution of identified cell populations is shown in Supplementary Figure S3A. The proportion of monocyte subsets is shown in Supplementary Figure S3B and differentially expressed genes are shown in Supplementary Table S2.

### 3.2 Cell-cell interactions in gout flare and severe COVID-19

**FIGURE 3:**
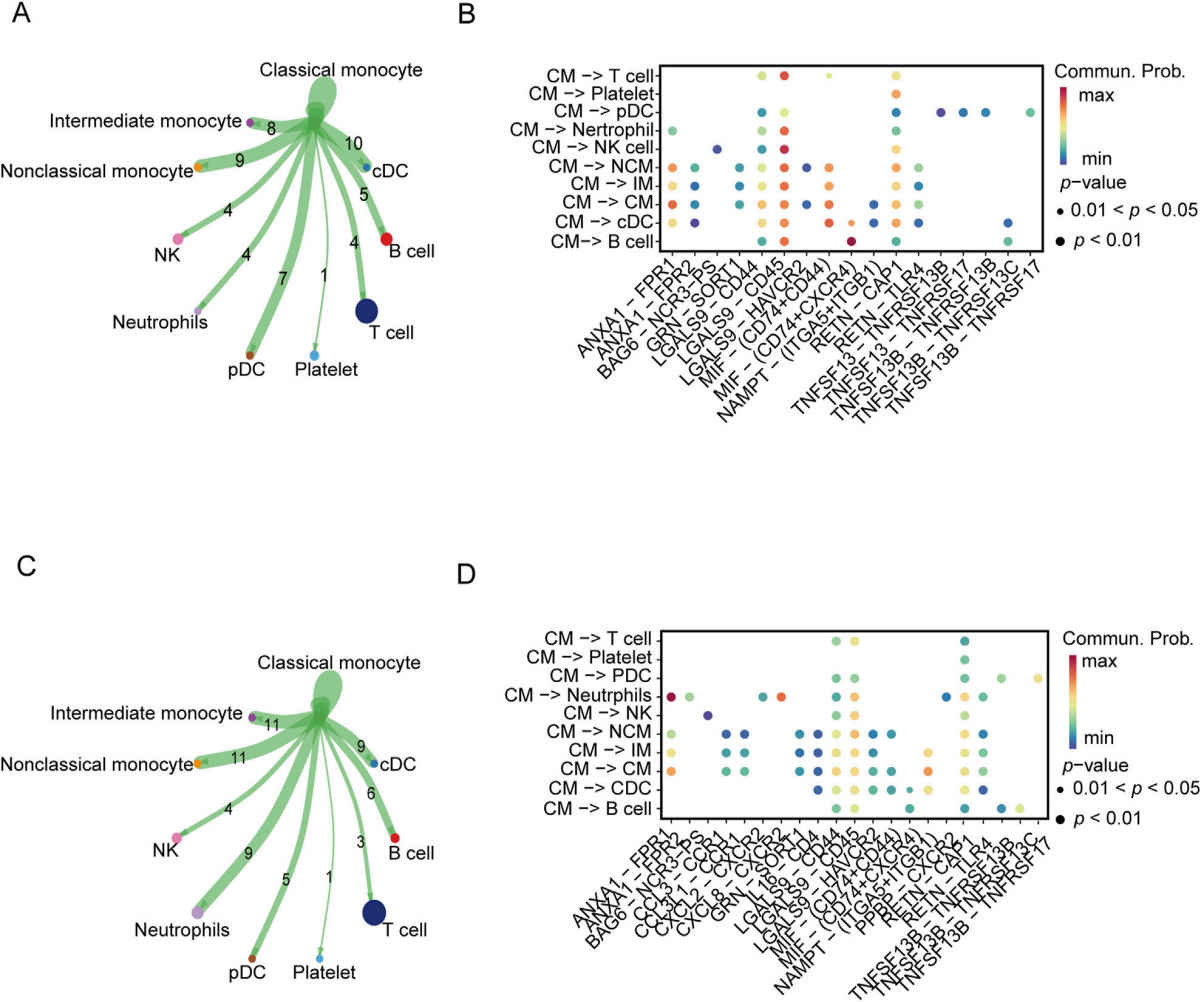
Cellular communication analysis in severe COVID-19 and gout. **(A, C)** Cellular communication networks constructed for classical monocyte and other cells in COVID-19 and gout samples, respectively. **(B, D)** Differential enrichment signaling pathway analysis between classical monocyte and other cells.

In the following analysis, we continued to focus on the association between severe COVID-19 and gout flares. The intercellular communication was analyzed by the CellChat algorithm. In the severe COVID-19 datasets, the CMs showed high interaction with IMs, NCMs, cDCs, and pDCs (Figure 3A). On the other hand, the major interaction partners of CMs in gout flare were IMs, NCMs, cDCs, and neutrophils (Figure 3C). We also identified several ligand-receptor pairs, and found that LGALS9 and CD45 were the predominant interacting pair in both diseases. RETN and CAP1 mediated the interactions between CM and the other cell subsets. In addition, MIF and (CD74+CXCR4) mediated significant interactions between CMs and B cells in the COVID-19 samples (Figure 3B). In the gout dataset, CMs and neutrophils showed strong interaction via ANXA1 and FPR1(Figure 3D).

### 3.3 Identification of genes related to severe COVID-19 and gout risk

**FIGURE 4:**
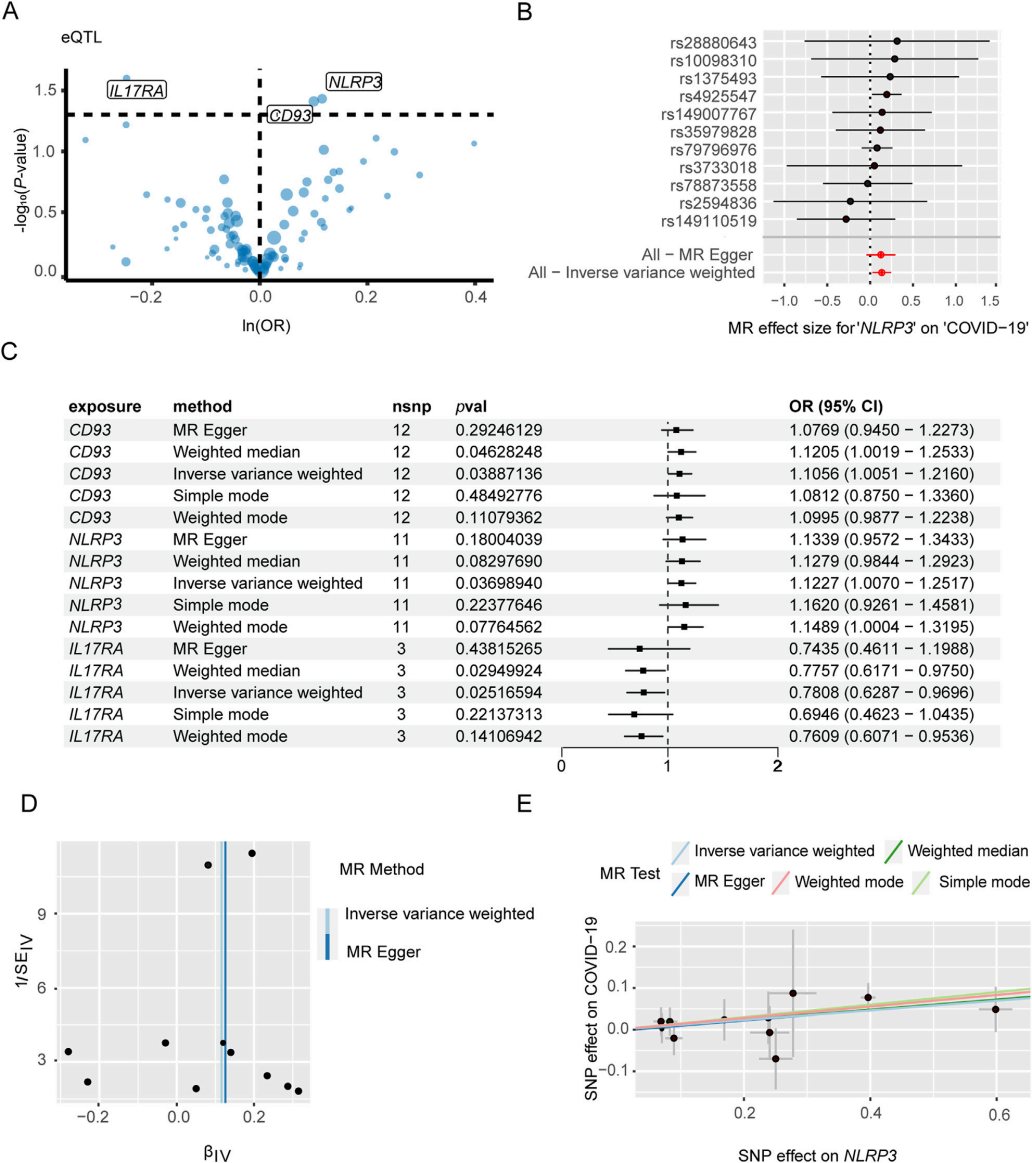
Mendelian randomization analysis of key genes and severe COVID-19. **(A)** Volcano plot illustrates the association between key genes and the risk of severe COVID-19. **(B)** Forest plot to visualize causal effect of each single SNP on the risk of severe COVID-19. **(C)** Mendelian randomization analysis shows associations of NLRP3, CD93, and IL17RA gene variants with severe COVID-19 risk. **(D)** The funnel plot indicates the effect of key genes on the risk of severe COVID-19. **(E)** Scatter plot demonstrates the effect of SNPs with exposure and outcome.

**FIGURE 5:**
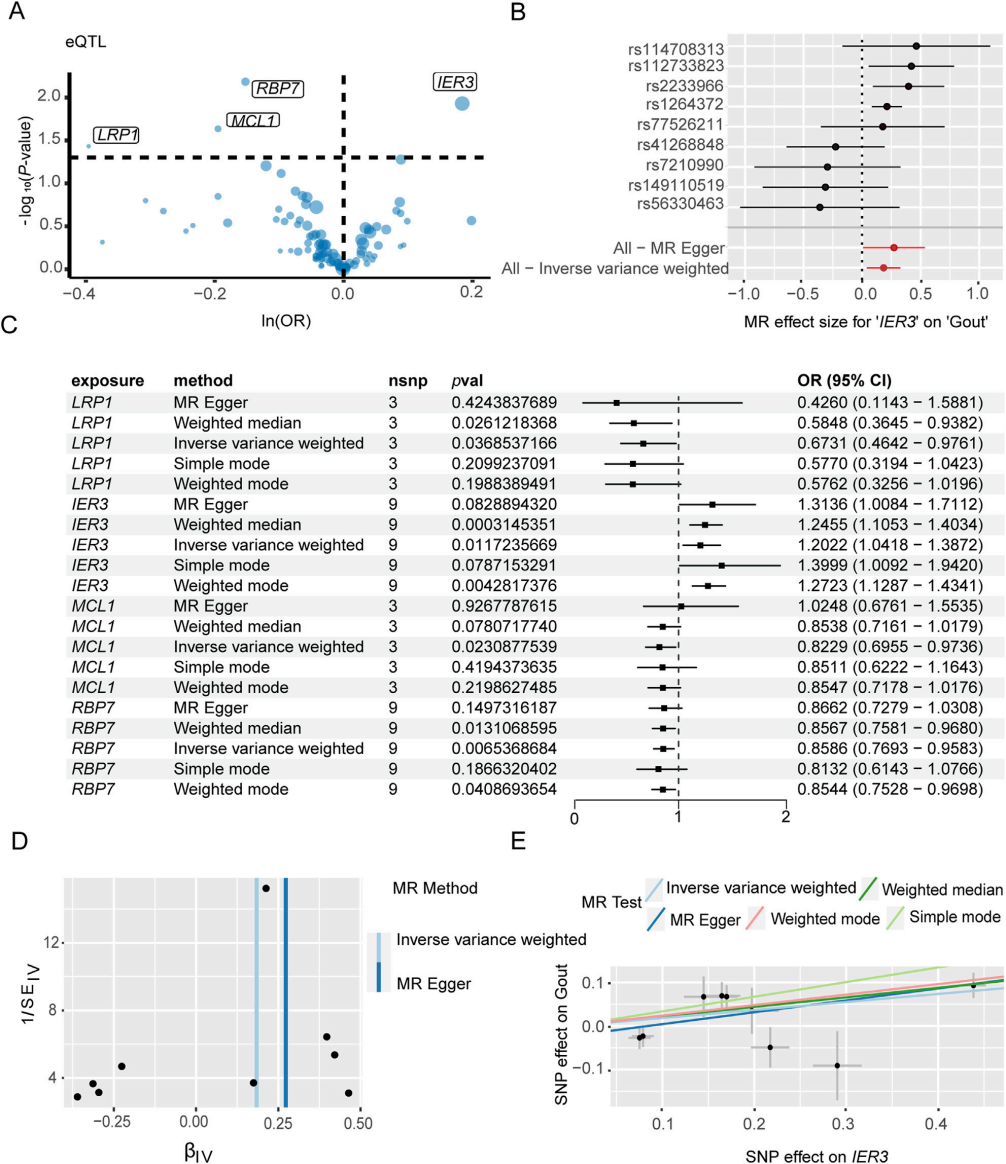
Mendelian randomization analysis of key genes and gout flares. **(A)** Volcano plot illustrates the association between key genes and the risk of gout flares. **(B)** Forest plot illustrates the effect sizes for the associations. **(C)** Mendelian randomization analysis shows associations of IER3, LRP1, MCL1, and RBP7 gene variants with the risk of gout flares. **(D)** The funnel plot indicates the effect of key genes on the risk of gout flare. **(E)** Scatter plot demonstrates the effect of SNPs with exposure and outcome.

The genes associated with severe COVID-19 infection and gout risk were identified by MR analysis as described in the methods. The volcano map highlights genes associated with COVID-19 that have significant *p*-values (Figure 4A). Forest plot shows the effect of *NLRP3*-associated SNPs on the risk of COVID-19 (Figure 4B). As shown in Figure 4C, higher expression levels of *NLRP3* and *CD93* were associated with an increased risk of severe COVID-19, whereas *IL17RA* was negatively correlated with severe COVID-19 risk. *NLRP3* showed the association with the risk of severe COVID-19 (OR 1.12, 95% CI 1.01–1.25, *P* = 3.70 × 10^−2^). The funnel plot was drawn to assess heterogeneity (Figure 4D), and scatter plot of SNP effects on exposure and outcome is shown in Figure 4E. Furthermore, 4 genes were significantly associated with gout (Figure 5A). Forest plot shows the effect of *IER3*-associated SNPs on the risk of gout (Figure 5B). *IER3* was found a positive correlation, whereas *LRP1*, *MCL1* and *RBP7* were negatively correlated with the risk of gout (Figure 5C). *IER3* showed the association with gout risk (OR 1.2, 95% CI 1.04–1.39, *P* = 1.17 × 10^−2^). The scatter plot and funnel plot are shown in Figures 5D, E. MR-Egger test did not show evidence of multiplicity of effects in both diseases (Supplementary Table S3; Supplementary Table S4). Cochran^’^s Q-test results showed no heterogeneity concerning severe COVID-19 but the analysis of gout showed heterogeneity. The results are detailed in Supplementary Tables S5, S6. We used an online tool (https://sb452.shinyapps.io/power) to calculate the minimum sample size required for this MR analysis. Calculations suggested the minimum sample size for COVID-19 and gout is 2,387,400 and 39,900, respectively, to reach 80% power.

**FIGURE 6:**
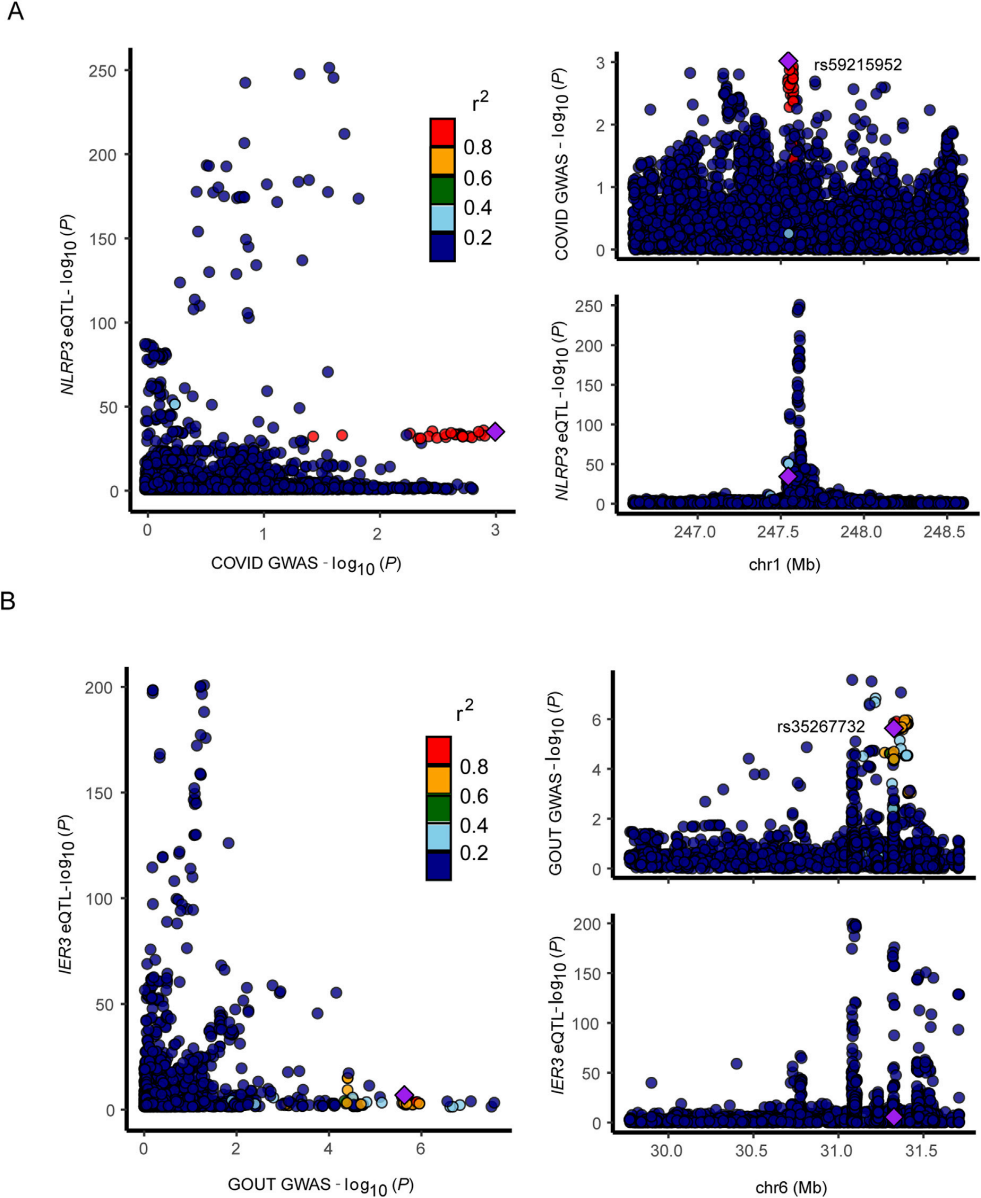
Regional association map. **(A, B)** Regional association plot depicts correlation of SNPs within specific genomic regions with gene and disease.

We selected the SNP with most significant association with *NLRP3* and *IER3* as a representative for co-localization analysis. Colocalization analyses between *NLRP3* and severe COVID-19 showed, PPH3 = 0.31, PPH4 = 0.11, and PPH3+PPH4 = 0.42. Colocalization analyses between *IER3* and gout showed, PPH3 = 0.99, PPH4 = 0.00, and PPH3+PPH4 = 0.99. *NLRP3* and severe COVID-19 were possibly associated with SNP loci in the genomic region, but were affected by different causal variants. On the other hand, *IER3* and gout were significantly associated with SNP loci in the genomic region, and were affected by different causal variants. The genetic variants near the region of *NLRP3* association with COVID-19 were shown in the regional association plot (Figure 6A), and the lead SNP was rs59215952. The regional association plot between *IER3* and gout is shown in Figure 6B, and the lead SNP was rs35267732. We also performed SMR analysis to verify the causal relationship between *NLRP3* and severe COVID-19, and the causal relationship between *IER3* and gout. The results showed that the two probes of *NLRP3* gene were ILMN_2310896 (*P*_SMR_ = 0.1324, *P*_HEIDI_>0.05, β = 0.1325, OR = 1.1417), and the topSNP was rs12143966; and ILMN_1712026 (*P*_SMR_<0.05, *P*_HEIDI_>0.05, β = 0.1822, OR = 1.2000), and the topSNP was rs10925027. Meanwhile, we found that the expression of *IER3* gene was associated with gout and there was no significant heterogeneity in the eQTL signaling (*P*_SMR_<0.05, *P*_HEIDI_>0.05, β = 0.0970, OR = 1.1018). The SMR results are shown in Table 1.

**TABLE 1:** SMR analysis and Heidi test results.

Ch	Gene	Gene probe	Top SNP	*P*_SMR_	*P*_HEIDI_	BE_SMR_	OR
		Probe position	SNP position		N_HEIDI_	SE_SMR_	95% CI
1	*NLRP3*	ILMN_2310896	rs12143966	0.1385	0.77	0.1325	1.1417
		247611978	247601357		20	0.0894	0.97–1.32
		ILMN_1712026	rs10925027	0.0477	0.7832	0.1822	1.1998
		247612006	247612562		20	0.092	1.10–1.38
6	*IER3*	ILMN_1682717	rs2233980	0.0092	0.055	0.097	1.1018
		30711220	31079644		20	0.0373	1.03–1.18

### 3.4 Cellular distribution of genes related to severe COVID-19 and gout flare

**FIGURE 7:**
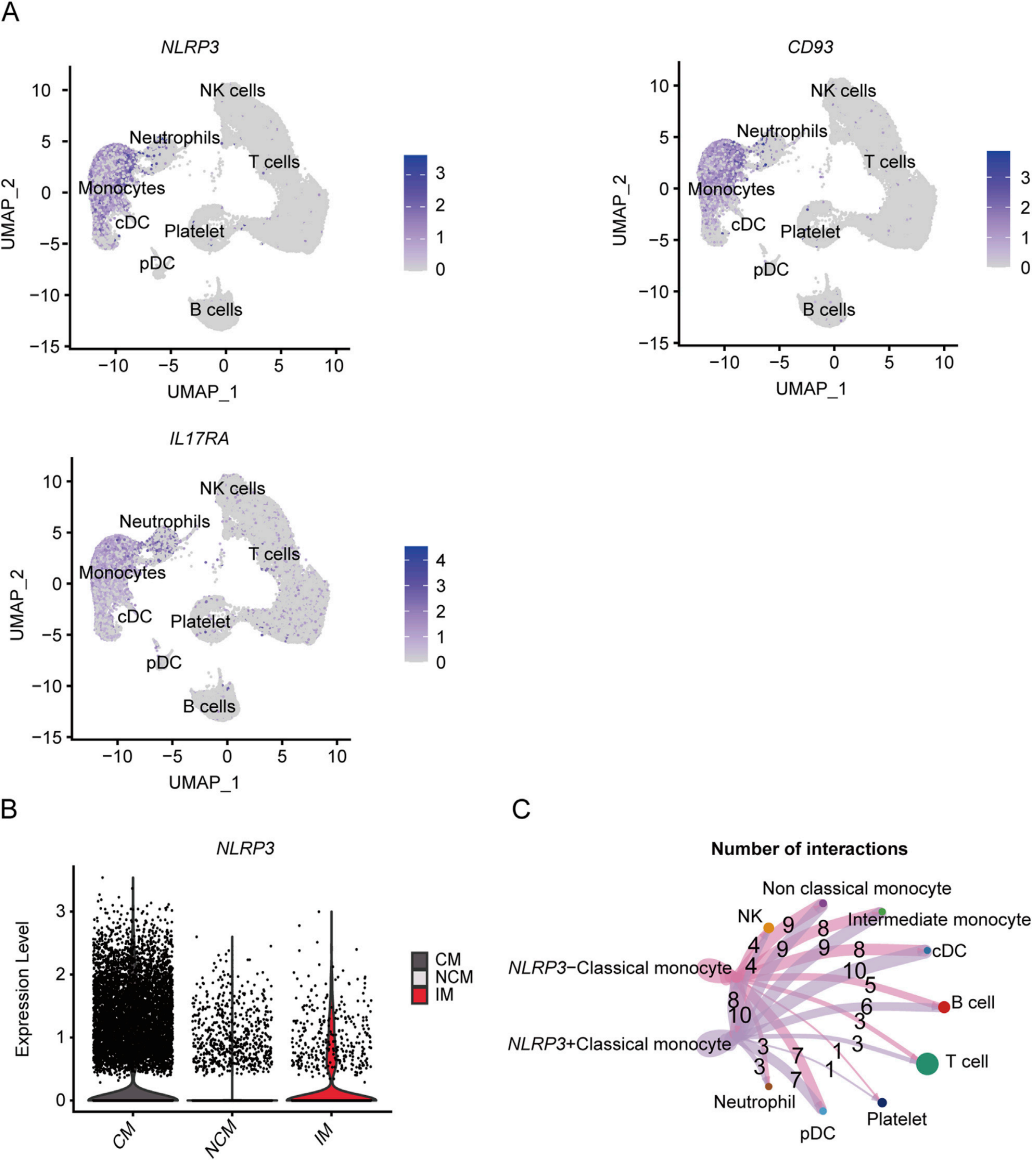
Expression of key genes associated with severe COVID-19 in different subsets and analysis of cellular communication between different cell susets. **(A)** The UMAP plot shows the distribution of key genes concerning severe COVID-19 in different cell subsets. **(B)** Violin plot shows the expression level of NLRP3 in CM, IM and NCM. **(C)** Cellular communication networks between NLRP3+CM and NLRP3-CM cell subgroups in COVID-19 samples.

**FIGURE 8:**
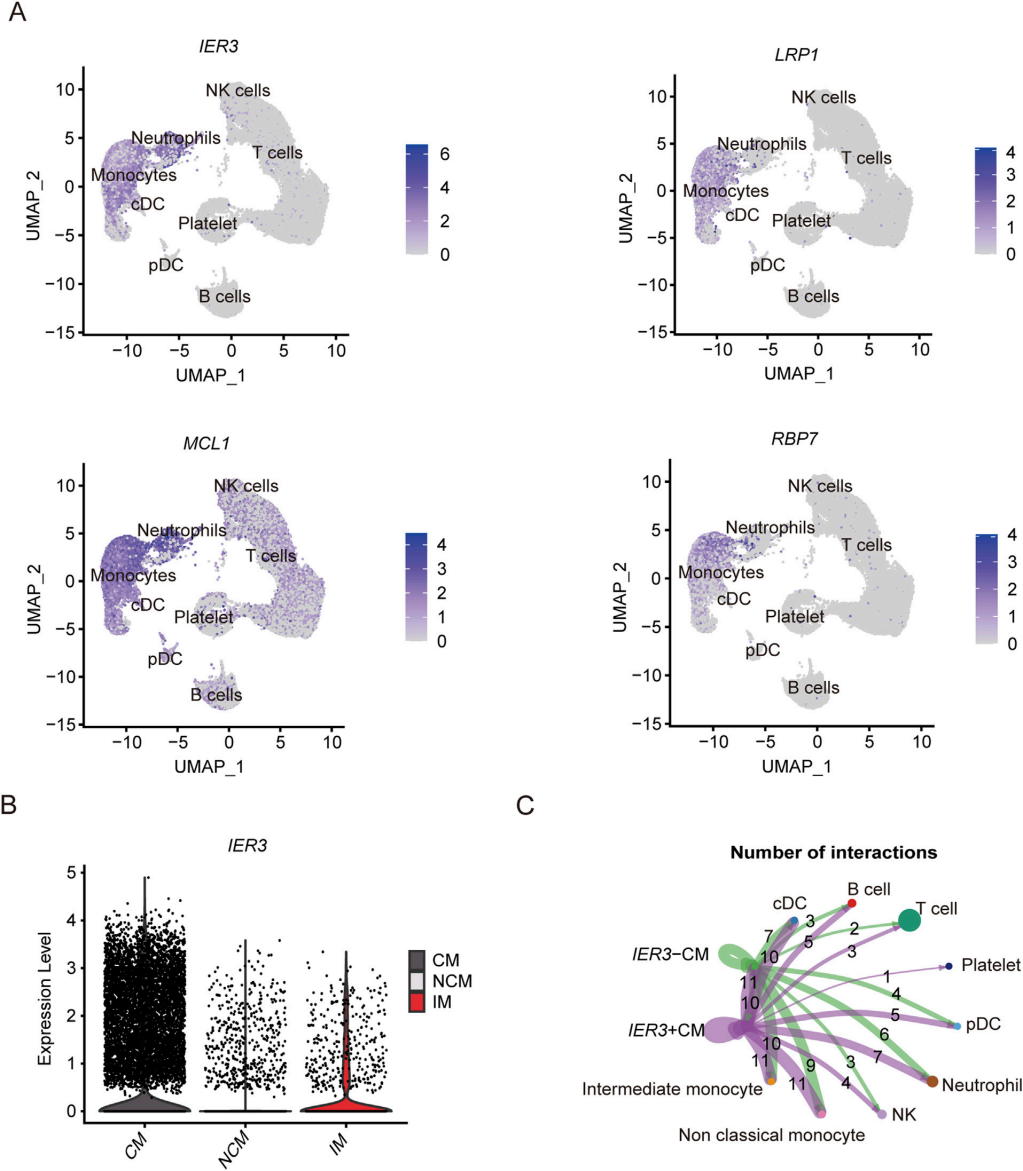
Expression of key genes associated with gout flares in different subsets and analysis of cellular communication between different cell susets. **(A)** The UMAP plot shows the distribution of key genes concerning gout flare in different cell subgroups. **(B)** Violin plot shows the expression level of IER3 in CM, IM and NCM. **(C)** Cellular communication networks between IER3+CM and I*ER3*-CM cell subgroups in gout samples.

As shown in the UMAP plot (Figure 7A), the monocytes expressed higher levels of *CD93*, *NLRP3*, and *IL17RA* compared to the other cell populations. Furthermore, *NLRP3* was highly expressed in the CMs, and showed lowest expression levels in the NCMs (Figure 7B). The network of interactions between the different subsets further showed strong interaction of the CMs with IMs, NCMs and cDCs (Figure 7C). The genes related with gout including *IER3*, *LRP1*, *MCL1* and *RBP7*, were predominantly expressed in monocytes (Figure 8A), of which *IER3* was expressed at higher levels in CMs compared to the other cell populations (Figure 8B). The network of interactions was shown in Figure 8C.

### 3.5 Key pathways related to severe COVID-19 and gout flare

**FIGURE 9:**
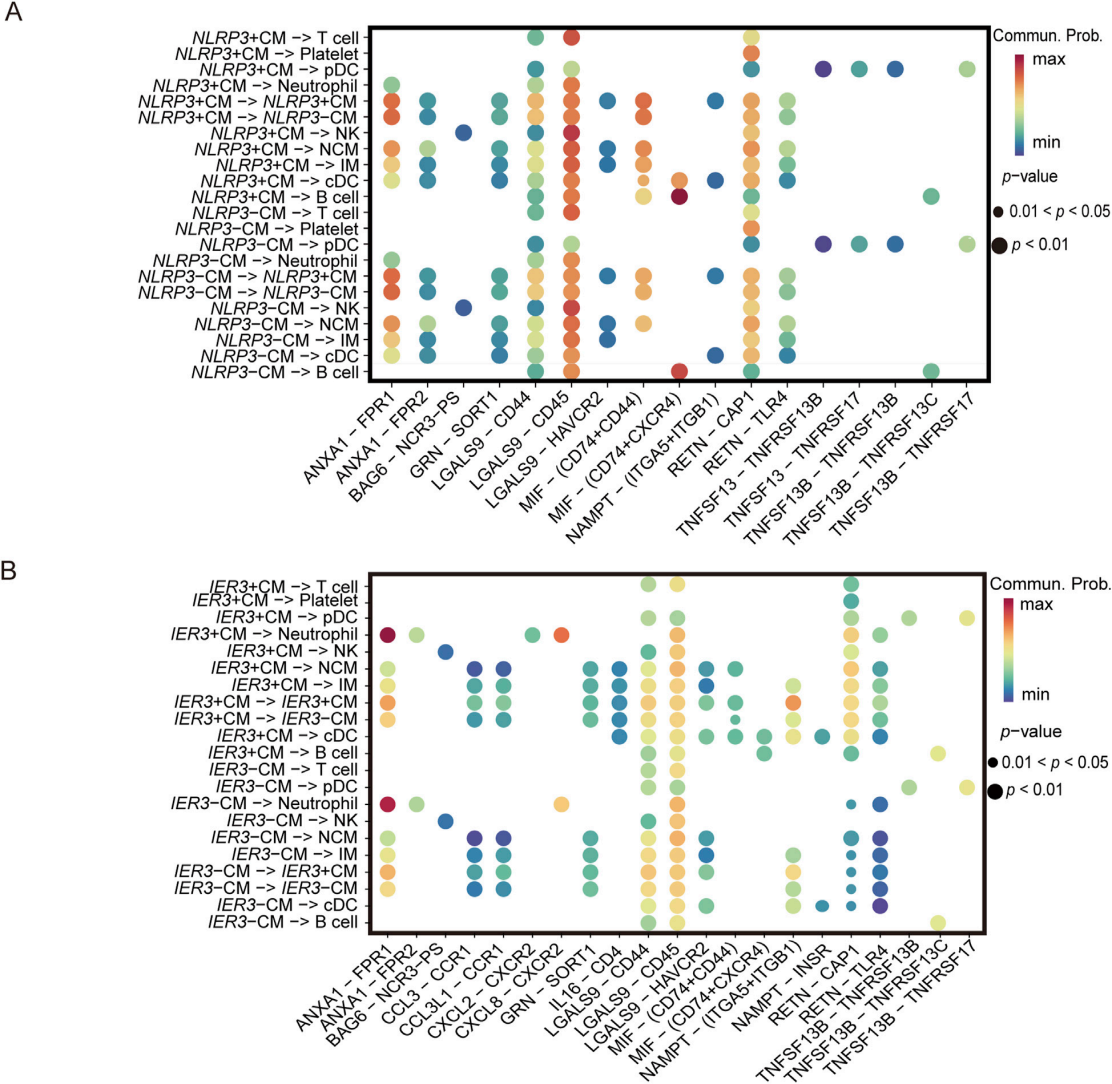
Ligand-receptor interactions identified in scRNA-seq data. **(A)** Differential signaling pathways enriched in communications involving NLRP3+CM cells compared to others. **(B)** Differential signaling pathways enriched in communications involving IER3+CM cells compared to others.

Results of the ligand-receptor interaction analysis showed that the *NLRP3*-positive CMs had more inter-cellular interactions compared to the *NLRP3*-negative CMs (Figure 9A). Furthermore, MIF and (CD74^+^CD44), MIF and (CD74+CXCR4) showed more cell-to-cell interactions. As shown in Figure 9B, the *IER3*-positive CMs showed more interactions than *IER3*-negative CMs involving MIF and (CD74+CD44), MIF and (CD74+CXCR4), RETN and CAP1, and RETN and TLR4 in gout flare. As a multifunctional cytokine, MIF mainly interacts with the CXC family of receptors (such as CXCR2, CXCR4, CXCR7, CXCR12), CD74, and CD44 to activate the downstream signaling pathways.

### 3.6 Metabolic profiles of the different cell populations

**FIGURE 10:**
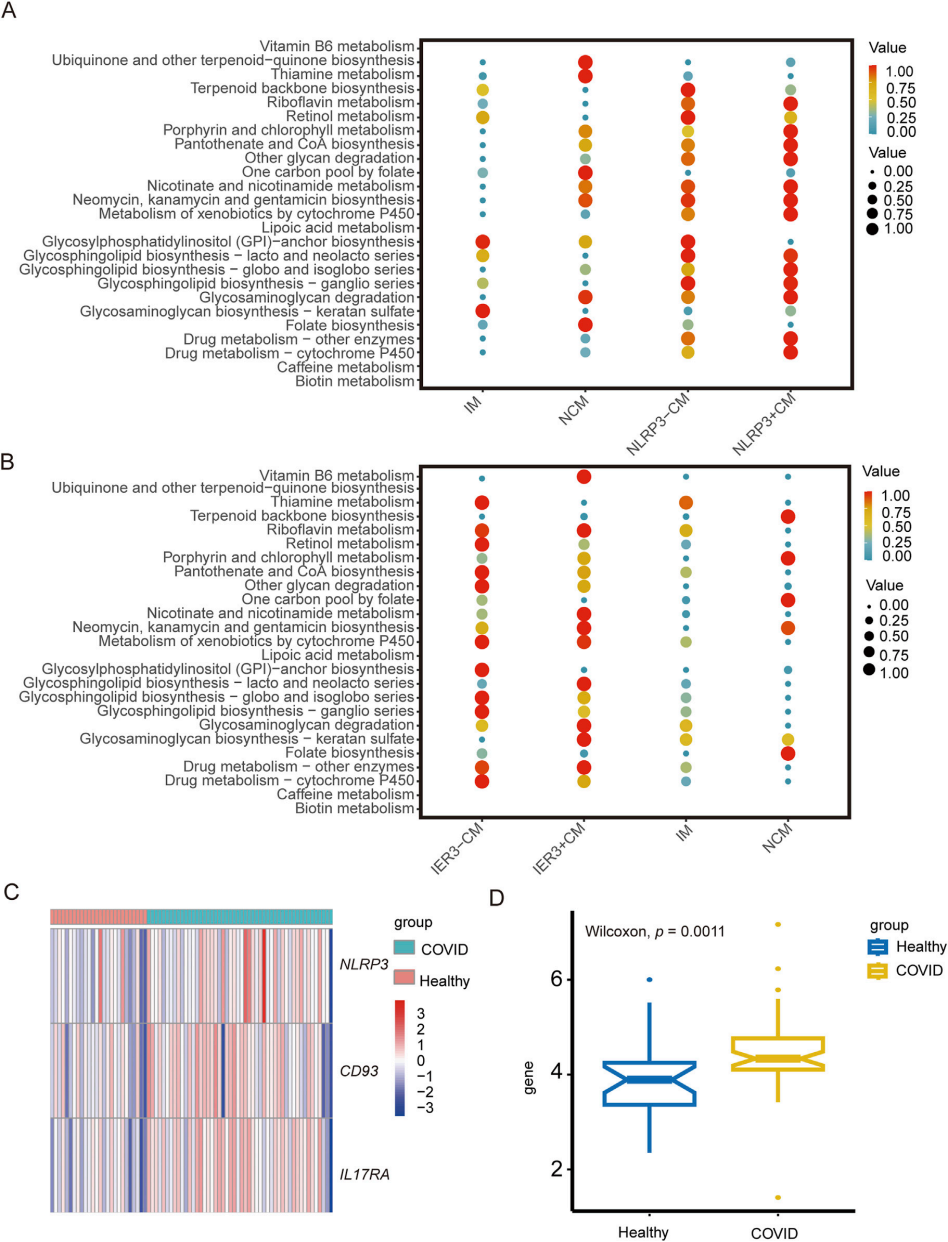
The role of *NLRP3* and *IER3* in monocyte metabolism and the difference of key genes expression in COVID-19 between different groups. **(A)** Metabolic pathway analysis reveals differences in metabolic activities and metabolic pathway between NLRP3+CM and NLRP3-CM cell subgroups. **(B)** Metabolic pathway analysis reveals differences in metabolic activities and metabolic pathway between IER3+CM and IER3-CM cell subgroups. **(C)** Heat map shows the expression levels of key genes in severe COVID-19 and healthy groups. **(D)** Box plot indicates significant differential expression of NLRP3 in severe COVID-19 and healthy groups.

The metabolic activities in the cell populations were identified using scMetabolism. Notably, glycosphingolipid biosynthesis-related pathways, riboflavin metabolism and cytochrome P450-related metabolic pathways were enriched in the CMs. Sphingolipids are indispensable components of cell membranes, and not only maintain membrane stability but also regulate cell adhesion, proliferation, apoptosis, differentiation, and recognition. Sphingolipids can act as exogenous or endogenous ligands for some immune cells and stimulate the secretion various cytokines, including TNF, IL-6, and IL-8, and therefore play key roles in inflammation-related and autoimmune diseases. Riboflavin plays a critical role in mitochondrial energy metabolism, redox homeostasis, cell apoptosis and inflammatory response. Cytochrome P450 is widely involved in pathological processes such as apoptosis, inflammatory response, oxidative stress and endoplasmic reticulum stress. *NLRP3*-positive CMs showed higher porphyrin and chlorophyll metabolism in the COVID-19 patients (Figure 10A), while the glycosaminoglycan biosynthesis-keratin sulfate pathway was enriched in the *IER3*-positive CMs in gout patients (Figure 10B). In contrast, the *IER3*-negative and *NLRP3*-negative CMs showed increased biosynthesis of glycosylphosphatidylinositol-anchored proteins (GPI-Aps). These results are indicative of the metabolic differences between the *NLRP3/IER3*-positve and *NLRP3/IER3*-negative CMs. Furthermore, we created a heat map to illustrate the levels of *NLRP3*, *CD93* and *IL17RA* genes expression by using bulkRNA-seq data from COVID-19 and healthy samples (Figure 10C). The bulk RNA-seq data confirmed the significantly higher expression of *NLRP3* in the severe COVID-19 patients compared to the healthy samples (Figure 10D), indicating that *NLRP3* plays a key role in disease progression.

## 4 Discussion

This study integrated GWAS and scRNA-seq data to identify cell types and key genes associated with gout flares and severe COVID-19. Our results showed that classical monocytes play a critical role in the progression of gout flare and severe COVID-19, and may involve the genes including *NLRP3* and *IER3*.

First, we analyzed scRNA-seq data from COVID-19 and gout to verify the presence of monocytes, T cells, DCs, etc. in peripheral blood. Among them, monocytes play an integral role in the immune response, but the role of monocytes in the interaction of gout and severe COVID-19 has not been established. Therefore, we focused on the relationship between monocytes and these two diseases. Monocytes originate from the hematopoietic stem cells and develop in the bone marrow, and play a central role in immune responses and host defense by activating lymphocytes, eliminating pathogens, and promoting tissue repair (Jakubzick et al., 2017; Merad and Martin, 2020; Qin et al., 2021). Several studies have shown that monocytes play an important role in the development of severe COVID-19 and gout (Amrute et al., 2022; Yu et al., 2023). For instance, SARS-CoV-2 infects monocytes via ACE2-dependent and ACE2-independent pathways, and triggers the release of large amounts of chemokines and inflammatory mediators (Jafarzadeh et al., 2020; Selva and Chung, 2022). In addition, gout flares are accompanied by the massive recruitment of circulating monocytes to the inflamed region, wherein they differentiate into pro-inflammatory macrophages and exacerbate tissue inflammation (Qadri et al., 2024). Monocytes are classified into three distinct subgroups: classical, intermediate, and nonclassical. Classical monocytes contribute to the pro-inflammatory defense mechanisms, intermediate monocytes are associated with antigen presentation, and non-classical monocytes mediate vascular patrolling and surveillance (Hally et al., 2022). Since pro-inflammatory cytokines like TNF and IL-1β are upregulated in the CMs of severe COVID-19 and gout patients, these cells likely contribute to the inflammatory response during gout flares and severe COVID-19. In the present study, we observed a significant increase in the proportion of CM subsets in severe COVID-19 and gout samples, which is consistent with previous findings. This suggests that an aberrant increase in the proportion of CMs is associated with the development of severe COVID-19 and gout.

There are many intersections between COVID-19 and gout flares. This study reveals the upregulated cell subsets in both diseases and further explores the characteristic pathways of each cell subset. Intercellular communication analysis showed that LGALS9 and CD45 were the predominant interacting pair in both diseases. LGALS9 is expressed in various cell types and mediates proliferation, differentiation, inflammation and formation of immune cells. LGALS9 and CD45 are significantly upregulated in rheumatoid arthritis patients (Zhang and Lee, 2022). Furthermore, RETN and CAP1 mediated the interactions between CM and the other cell subsets. CAP1 is a functional receptor of human resistin that can regulate the inflammatory response. Sun et al. (2023) showed that the RETN-CAP1 signaling pathway was activated in the C10_ULK1 cell cluster identified in patients with *Escherichia coli* sepsis, and these cells promoted systemic inflammation by secreting RETN.

To further investigate the role of core subsets, we identified the DEGs of CMs. DEGs were used for MR analysis and key genes causally associated with disease were identified. *NLRP3* and *IER3* have the highest ORs in key genes associated with COVID-19 and gouty flares, respectively. To ensure the reliability of the results, we performed co-localization and SMR analyses. The results of MR, SMR and Colocalization analysis showed that *NLRP3* and *IER3* were causally associated with the risks of COVID-19 and gout flare. *NLRP3* was positively correlated with the risk of severe COVID-19, and *IER3* was positively correlated with gout, although the analysis results were not significant after correction by Bonferroni method. Given this paper is an exploratory study, the potential association evidence is also valuable for further exploration of the disease. On the other hand, since the effect of genotype on phenotype is usually small, Mendelian randomization analysis may require a very large sample size to achieve sufficient effects. The calculated minimum sample size was 2,387,400, while the actual sample size collected was 1,388,342 in the study of COVID-19. Therefore, it may lead to a lack of significance in the results. Large-scale studies are still needed for further verification. The bulk RNA-seq data of severe COVID-19 patients also indicated significant *NLRP3* upregulation. This further validates the results of our analysis.

The *NLRP3* inflammasome is mainly expressed in peripheral macrophages, monocytes and dendritic cells. Excessive inflammasome activation may be involved in the development of gout, sepsis, type 2 diabetes, atherosclerosis, neurological disorders, and other inflammation-related diseases (Sharma and Kanneganti, 2021). *NLRP3* inflammasome consists of a sensor protein (NLR family PYRIN structural domain containing-3, NLRP3), a junction protein (apoptosis-associated speck-like protein, ASC), and effector proteins (caspase-1) (Kelley et al., 2019). Upon receiving danger signals such as infection, NLRP3 recruits and activates pro-caspase-1 via ASC, and the activated caspase-1 cleaves IL-1β and IL-18 precursors into the active pro-inflammatory forms (Fu and Wu, 2023). IL-1β then stimulates the release of inflammatory factors such as TNFα, IL-6, and IL-8, which can lead to a “cytokine storm” in acute inflammatory diseases (Pan et al., 2021). The *NLRP3* inflammasome is activated in mononuclear phagocytes by exogenous stimuli ranging from crystalline microparticles to viral proteins, including SARS-CoV virus channel protein, hepatitis C virus core protein, and influenza virus M2 protein (Honda et al., 2023). Gout is also an *NLRP3* inflammasome-associated disease and involves multiple inflammatory cytokines (Wu et al., 2020). Overall, *NLRP3*-induced inflammation is a key driver of the development of COVID-19 and gout, and may play an important role in the mutual promotion of both diseases. Therefore, inflammasome inhibitors can potentially prevent or slow down disease progression.

*IER3* (immediate early response 3) is a stress-inducible gene involved in the pathogenesis of multiple diseases, and can be rapidly induced in response to viral infections, inflammatory cytokines, chemical carcinogens, and other types of stimuli (Wu et al., 2013). The *IER3*-knockout mice develop persistent hypertension with little signs of vascular or renal inflammation, indicating that *IER3* may be involved in inflammation (Shahid et al., 2010). Moreover, *IER3* deficiency impairs the ability of macrophages and T cells to respond to stimuli (Shahid et al., 2018). *IER3* excerts effects in regulating cellular proliferation and apoptosis, for instance, overexpression of *IER3* in T cells inhibited apoptosis and increased susceptibility to lupus-like autoimmune diseases (Arlt and Schafer, 2011). Furthermore, the absence of *IER3* gene can reduce inflammatory responses and induce an increased cell apoptosis mediated through a reduction in VEGF-A/MCP-1 axis and MMP-9 (Brahmbhatt et al., 2014). On the other hand, *IER3* plays a pro-apoptotic role in various tumors, ischemic acute kidney injury, and other diseases (Morinobu et al., 2016; Tang et al., 2023). Apoptosis is involved in the pathogenesis of gouty arthritis, and has been observed in macrophages of gouty stones from GA patients (Chen et al., 2023). Furthermore, MSU crystal-induced removal of downstream apoptotic inflammatory cells can indirectly abort acute gout attacks (Steiger and Harper, 2014). Inhibition of monocyte apoptosis by *IER3* prolonged the response to MSU crystallization-induced inflammation, which in turn promoted their differentiation into macrophages, resulting in greater monocyte recruitment and tissue damage. COVID-19 development has also been linked to apoptotic mechanisms (Chu et al., 2021; Pan et al., 2023). Nevertheless, the exact role of *IER3* in gout and severe COVID-19 remains to be elucidated further.

Finally, according to the expression of *NLRP3* and *IER3*, CMs was divided into two subgroups: *NLRP3*/*IER3*-positive CMs and *NLRP3*/*IER3*-negative CMs. We identified cellular communication and assessed metabolic activities between *NLRP3*/*IER3*-positve CMs and other monocyte subsets. Differences in cellular communication between the *NLRP3*/*IER3*-positve and *NLRP3*/*IER3*-negative CMs were observed and involved the MIF signaling pathway. MIF usually bind to membrane receptors in the form of complexes (CXCR4/CD74, CXCR2/CD74, CD74/CD44) and regulate cellular functions. For example, the binding of MIF to CD74/CD44 complexes can regulate immune responses and trigger inflammation, tumors, and autoimmune diseases (Christopoulou et al., 2024; Ma et al., 2024; Zhao et al., 2023). Given that the pathogenesis of gout flare and severe COVID-19 involves inflammation and immune responses, we surmise that the MIF-related pathways play key roles in their occurrence and development. Meanwhile, the glycosphingolipid biosynthesis pathway, riboflavin metabolism and cytochrome P450-related metabolic pathways were enriched in CMs. These pathways could be related to the role of classical monocytes in inflammation regulation, infection control and tissue repair. Additionally, increased biosynthesis of glycosylphosphatidylinositol anchor-protein was observed in the *NLRP3*/*IER3*-negative CMs. The GPI-Aps are ubiquitous in eukaryote cells and mediate ligand recognition, enzyme activity, cell-to-cell interactions, host infection, and defense responses (Chun et al., 2022).

There are some limitations in this study that ought to be recognized. First, the sample size was relatively small, and further studies on larger cohorts are necessary. Second, we focused on PBMCs, which may not fully represent the local inflammatory and immune responses that occur during gout flares and remissions. Third, the Mendelian analyses involved data from a European population, which may limit the applicability of our findings to other populations. In addition, mendelian randomization is an emerging study strategy that reduces confounding and reverse causality but relies heavily on gene-level analysis whose reliability is still being tested.

## 5 Conclusion

In conclusion, our findings provide new insights into the pathogenesis of COVID-19 and gout from genetic and immunologic perspectives, along with potential biomarkers and therapeutic targets. Subsequent studies should be validated using multiple experimental methods, which may be helpful to guide the diagnosis and treatment of patients with gout and COVID-19.

## Data Availability

The original contributions presented in the study are included in the article/Supplementary Material, further inquiries can be directed to the corresponding author.

## Supplementary material

SUPPLEMENTARY FIGURE S1 The expression profiles of characteristic genes in different cell clusters. **(A)** Dot plot shows the proportion (point size) and average expression (color scale) of cell clusters identified in PBMC which express marker genes from COVID-19, gout flare and healthy samples. **(B)** Dot plot shows the proportion (point size) and average expression (color scale) of cell clusters identified in PBMC which express marker genes from COVID-19, gout remission and healthy samples. **(C)** Dot plot shows the proportion and average expression of cell clusters expressing the marker gene in monocytes from COVID-19, gout flare and healthy samples. **(D)** Dot plot shows the proportion and average expression of cell clusters expressing the marker gene in monocytes from COVID-19, gout remission and healthy samples.

SUPPLEMENTARY FIGURE S2 Identification of cell subpopulations. **(A)** Total cell subclusters and monocyte subclusters from severe COVID-19, gout flare, and healthy samples. **(B)** Total cell subclusters and monocyte subclusters from severe COVID-19, gout remission, and healthy samples.

SUPPLEMENTARY FIGURE S3 Single-cell analysis of severe COVID-19, gout remission and healthy samples. **(A)** UMAP plot displays the results after clustering. NK cells, T cells, B cells, pDCs, cDCs, monocytes, neutrophils, and platelets were identified. **(B)** Depicts the proportion of each monocyte subgroup in different samples.

SUPPLEMENTARY TABLE S1 List of DEGs between monocytes and other cell types from samples of severe COVID-19, gout flare and healthy controls.

SUPPLEMENTARY TABLE S2 List of DEGs between monocytes and other cell types from samples of severe COVID-19, gout remission and healthy controls.

SUPPLEMENTARY TABLE S3 The table shows the results of pleiotropy tests performed for key genes and severe COVID-19.

SUPPLEMENTARY TABLE S4 The table shows the results of pleiotropy tests performed for key genes and severe gout.

SUPPLEMENTARY TABLE S5 The table shows the results of heterogeneity tests performed for key genes and severe COVID-19.

SUPPLEMENTARY TABLE S6 The table shows the results of heterogeneity tests performed for key genes and gout.

## Abbreviations

MR, Mendelian randomization; SMR, Summary-data-based Mendelian randomization; IV, Instrumental variable; eQTL, Expression quantitative trait locus; GWAS, Genome-wide association study; HEIDI, Heterogeneity in dependent instruments; IVW, Inverse variance weighting; LD, Linkage disequilibrium; scRNA-seq, Single-cell RNA sequencing; SNP, Single nucleotide polymorphism; DEGs, Differentially expressed genes; MSU, Monosodium urate; COVID-19, Coronavirus disease 2019; GEO, Gene Expression Omnibus; GPI-Aps, Glycosylphosphatidylinositol-anchored proteins; PCA, Principal component analysis.
