# Supplementary figures and images for: Integrating transcriptomics, eQTL, and Mendelian randomization to dissect monocyte roles in severe COVID-19 and gout flare
Source: Front Genet. 2024 Sep 25;15:1385316. doi: 10.3389/fgene.2024.1385316 (PMC11461236; doi:10.3389/fgene.2024.1385316)

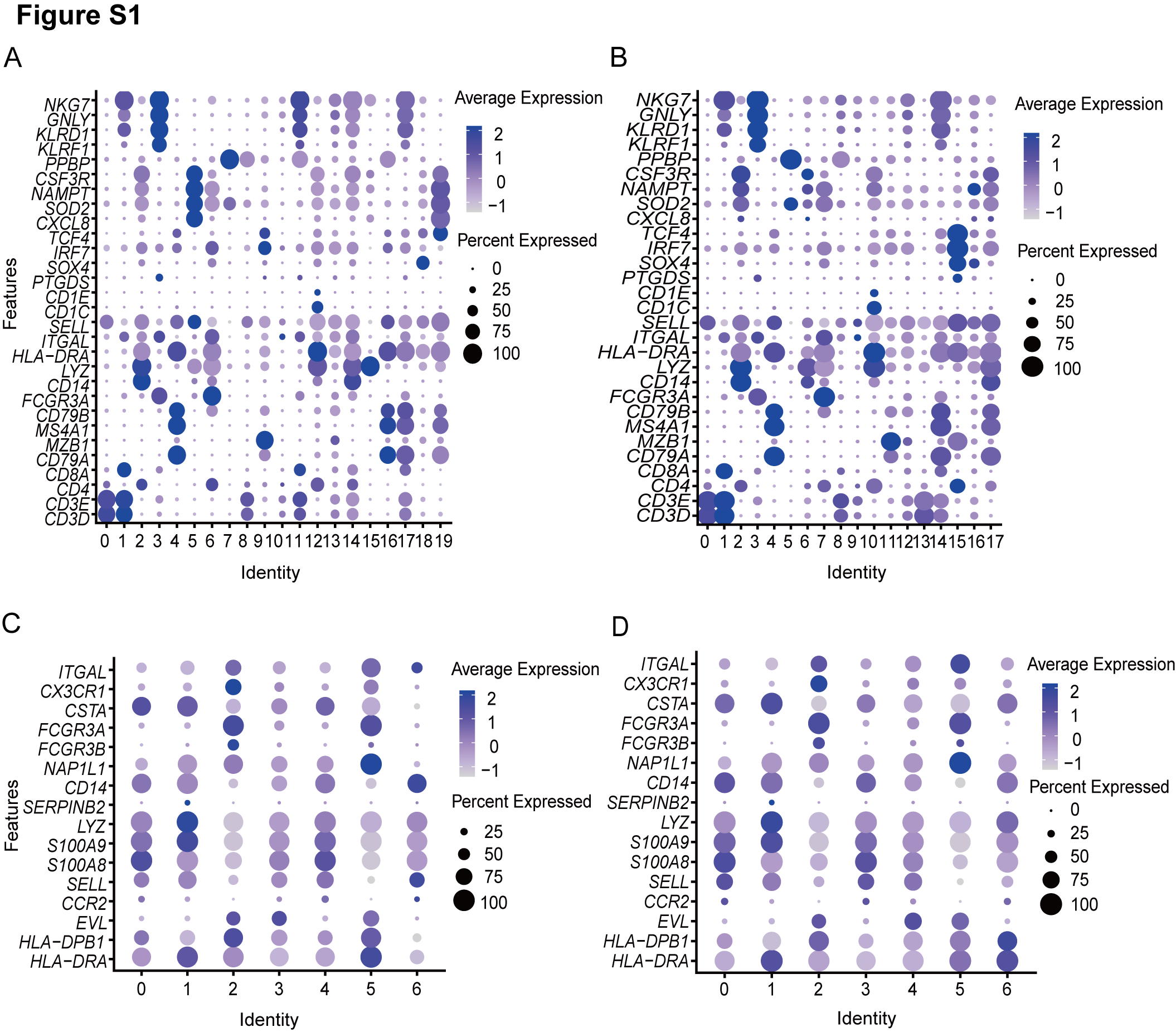

Supplement: Supplementary file 1 [file Presentation1.zip › Supplementary file/Figure S1.TIF]

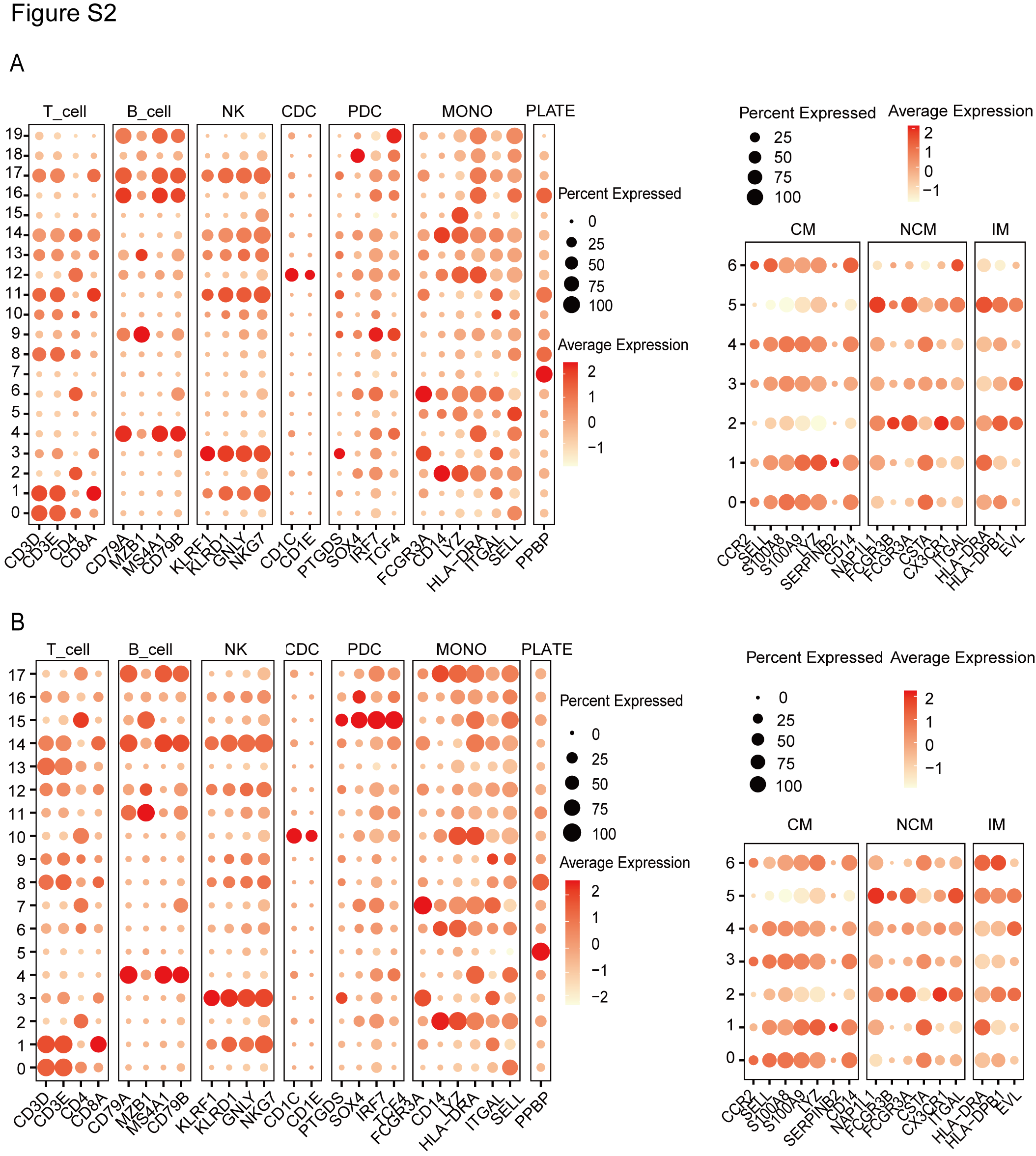

Supplement: Supplementary file 1 [file Presentation1.zip › Supplementary file/Figure S2.TIF]

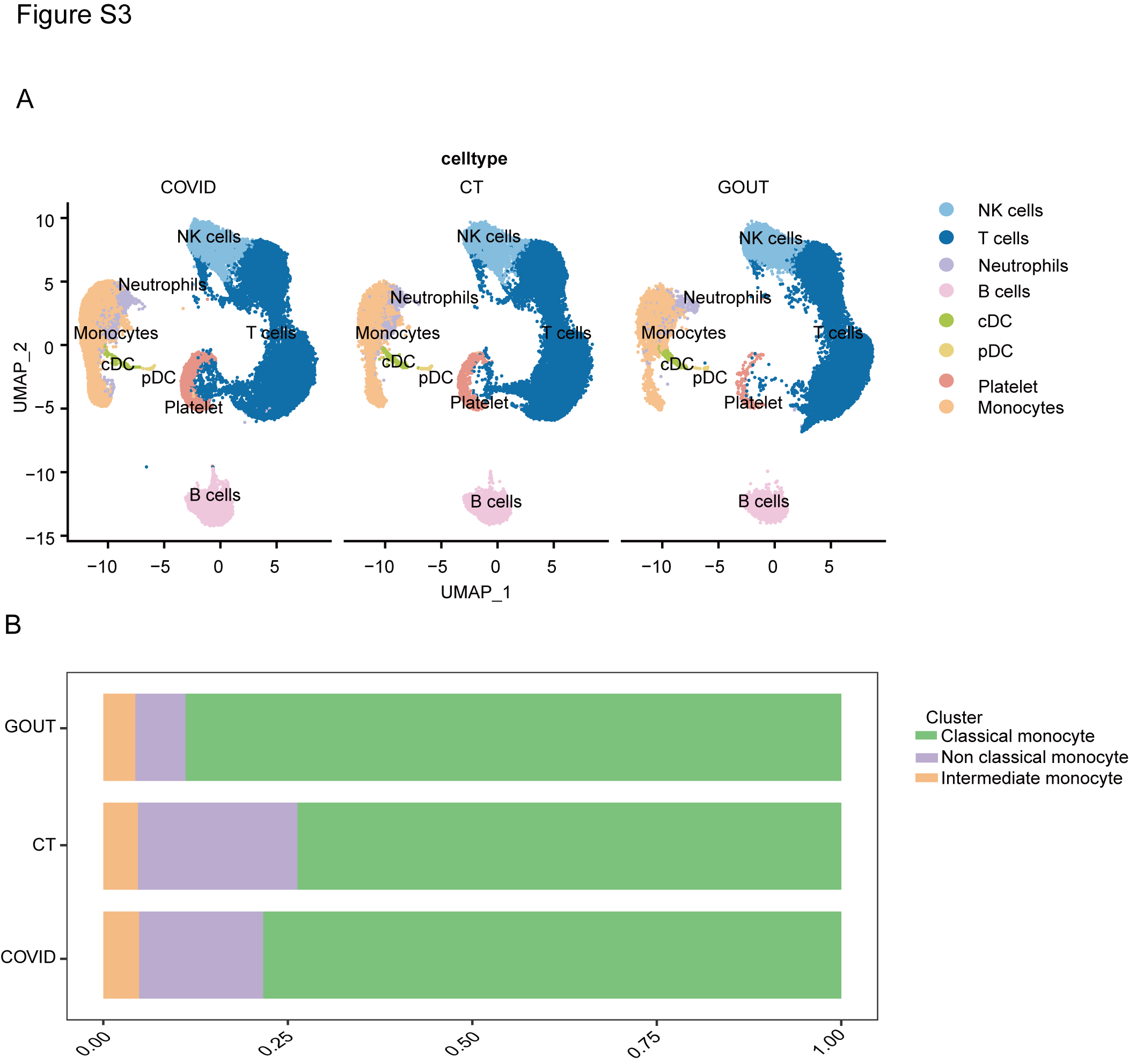

Supplement: Supplementary file 1 [file Presentation1.zip › Supplementary file/Figure S3.TIF]
